# The effect of non-pharmaceutical interventions on the demand for health care and on mortality: evidence from COVID-19 in Scandinavia

**DOI:** 10.1007/s00148-021-00868-9

**Published:** 2021-07-28

**Authors:** Steffen Juranek, Floris T. Zoutman

**Affiliations:** 1 NHH Norwegian School of Economics, Bergen, Norway; 2 NHH Norwegian School of Economics AND NOCET and CESifo, Bergen, Norway

**Keywords:** COVID-19, Non-pharmaceutical interventions, Healthcare costs, Mortality, Health economics, I10, I12, I18

## Abstract

We study the effectiveness of non-pharmaceutical interventions (NPIs) against COVID-19 on the allocation of scarce resources in the hospital sector in Scandinavia. Denmark and Norway imposed strict NPIs, but Sweden followed an extraordinarily lenient approach. We use an event study to compare COVID-19 hospitalizations, intensive-care (ICU) patients, and deaths in Sweden with Denmark and Norway. The outcome variables initially follow a common trend, but diverge 2–3 weeks after lockdown. Both the timing of the effect and the similarity in the trend between Denmark and Norway are highly consistent with a causal effect of the lockdown. We use our event study to build a counterfactual model that predicts the outcome variables for Denmark and Norway if they had followed Sweden’s approach. In the absence of strict NPIs, the peak number of hospitalizations would have been 2.5 (3.5) times as large in Denmark (Norway). Overall, Denmark (Norway) would have had 334 (671) percent more hospital-patient days, 277 (379) percent more ICU-patient days, and 402 (1015) percent more deaths. The benefit of lockdown in terms of healthcare and mortality costs amounts to between 1 and 4 (0.9 and 3.5) percent of GDP in Denmark (Norway).

## Introduction

COVID-19 puts a large burden on the healthcare system since many patients with COVID-19 require hospitalization or intensive care (ICU). From a health economics perspective, it is crucial to understand the extent to which non-pharmaceutical interventions (NPIs) affect the allocation of these scarce resources, and reduce the pressure on the hospital sector. This study provides direct causal evidence on the relationship between NPIs and the demand for hospital resources.

Our study provides evidence from the three Scandinavian countries Denmark, Norway and Sweden. Scandinavia forms an ideal laboratory to study this question. First, the three countries are similar in terms of culture, climate, healthcare and institutional framework. Second, community spread of COVID-19 started at the same time; Norway reported its 100th case on March 4, Sweden on March 6, and Denmark on March 9. Third, throughout the pandemic, the three countries have maintained high-quality comparable databases regarding hospitalizations and ICU admissions, thus allowing us to provide direct evidence from the hospital sector. Fourth, the NPIs vary strongly between the three countries. Denmark and Norway introduced strong NPIs early in the pandemic. On the other hand, Sweden imposed an extraordinary light NPI regime. The similarity between the three Scandinavian countries in all aspects except NPIs provides a unique source that allows us to isolate the causal effect of the NPIs on hospital resources.^1^

We use a difference-in-difference (DiD) event-study to analyze the effect of the lockdown in Denmark and Norway on the daily number of patients in hospitals with COVID-19, the daily number of patients in intensive-care (ICU) with COVID-19 and the daily cumulative number of COVID-19 deaths. Our main analysis covers the period March 5–June 30, 2020, which comprises the first wave of COVID-19 in Scandinavia.

We find that between the start of our sample period and 2–3 weeks into the lockdown period, the number of ICU patients and mortality per capita follow a common trend in the three countries.^2^ After, the outcome variables diverge rapidly, increasing in Sweden but plateauing and eventually decreasing in Denmark/Norway. Trends in Denmark and Norway are remarkably similar throughout the sample period. Both the timing of the effect and the similarity between Denmark and Norway are strongly indicative of the idea that we are measuring the causal impact of the NPIs.

Phasing out the lockdown in Denmark and Norway does not result in an increase in our outcome variables. However, from the end of April onward, the number of hospitalizations and ICU patients in Sweden decline, and hence, the DiD between Sweden and Denmark/Norway decreases as well.

We use our event-study model to predict the peak number of patients Denmark and Norway would have had in the counterfactual in which they did not lock down. Our model predicts that for Denmark (Norway) the peak number of hospitalizations would have been 144 (257) percent higher. The peak number of ICU patients would have been 103 (152) percent higher had Denmark (Norway) decided not to lock down.

We also consider the overall effect of the lockdown up to the end of our sample period. We find that in the counterfactual without lockdown, Denmark (Norway) would have had 334 (671) percent more hospitalization-patient days, and 277 (379) percent more ICU-patient days. Deaths would have been 402 (1015) percent higher.

Using conventional estimates for the cost of hospitalization and intensive care from the literature, we find that lockdown saved Denmark (Norway) 96 (94) million US dollars. We also consider mortality costs focusing on a scenario with low mortality cost and a scenario with high mortality cost. We find that the overall healthcare and mortality costs Denmark (Norway) saved through lockdown amounts to between 1 and 4 (0.9 and 3.5) percent of GDP.

The main policy conclusion is that the lockdown in Denmark and Norway has been extremely successful in reducing peak pressure on the healthcare system and reducing healthcare and mortality cost. Given that the duration of the lockdown was relatively short (phase-out started after 5 weeks), and given that the economic damage of the lockdown appears to have been relatively limited (see Sheridan et al.^2020^, Juranek et al. 2020), we conclude that the benefits of the lockdown outweigh the cost.

We also take a look at the long-run impact of the pandemic. In the second and third waves, a causal design is no longer plausible because the spread of COVID-19 does not follow the same trajectory across the three countries. However, our main conclusion is that Sweden has been most severely affected by all three COVID-19 waves. Moreover, likely related to the much higher case load, Sweden had to maintain a much stricter NPI regime throughout the remainder of 2020 and the beginning of 2021. There is thus no evidence that Sweden’s approach to COVID-19 during the first wave provides a long-term benefit.

One of the main contributions of our study is that we use data directly from the hospital sector, whereas most of the literature only focuses on the number of confirmed COVID-19 infections and/or mortalities (e.g., Courtemanche et al.2020b; Flaxman et al. 2020). Hospital data has several advantages. First, hospital beds, and specifically ICU beds, are likely to serve as a bottleneck in the healthcare system because a significant number of patients with COVID-19 require intensive care and supply in terms of personnel and equipment is limited in most countries. Therefore, from a health economics perspective, it is important to understand how NPIs affect these variables. Second, patients with COVID-19 who end up in hospitals are likely to be tested because testing is required to protect other patients and staff. Therefore, hospitalization data provides a more reliable source of information on the spread of COVID-19 than the number of confirmed cases which is in part driven by differences in the testing regime. Third, the economic costs of patients who end up in hospitals, ICUs or who pass away are arguably larger than less severe COVID-19 cases.

There is a rapidly growing literature that considers the effect of NPIs on the spread of COVID-19. We contribute in four ways. First, the Scandinavian setting offers clean identification because the countries are very similar, COVID-19 community spread occurred simultaneously in all three countries, and despite this, Sweden’s approach to COVID-19 differs from its neighbors. In contrast, in US (e.g., Courtemanche et al. 2020a, 2020b; Friedson et al. 2021; Allcott et al. 2020) and German (e.g., Glogowsky et al. 2020; Mitze et al. 2020) states, jurisdictions implement similar NPIs in rapid succession and timing is endogenous to the severity of the outbreak.^3^ Second, relative to the epidemiological literature (e.g., Flaxman et al. 2020) identification comes from the assumption that Sweden serves as a valid counterfactual to Denmark and Norway, rather than a structural epidemiological model. Third, we are the first empirical study to provide direct insights on the effect of NPIs on hospitalizations and intensive care admissions. Fourth, we are the first empirical study to provide a monetary quantification of the benefit of lockdown in terms of the reduction in healthcare expenditure and mortality.

## Institutional background

In this section, we discuss the differences in NPIs between the three Scandinavian countries, and provide a general comparison between the countries in terms of healthcare and demographics.

Table 1 displays the dates of the introduction of various measures based on the Oxford COVID-19 Government Response Tracker (Hale et al. 2020) during the first wave. As can be seen, Denmark and Norway introduced almost identical packages of NPIs. The only difference is that Norway also introduced restrictions on internal movement, whereas Denmark did not. On the other hand, in Sweden, primary schools, kindergartens and workplaces remained open throughout the first wave of the pandemic.^4^ A crucial reason why Sweden acted differently than its Scandinavian peers lies in the Swedish constitution (Jonung 2020). The Swedish constitution guarantees the freedom of movement and, therefore, favors recommendations over restrictions.

**Table 1:** Timing of the measures taken

Measure	Denmark	Norway	Sweden
School closing (some levels)	Mar 13–	Mar 12–May 10	Mar 17–
School closing (all levels)	Mar 13–Apr 14	Mar 12–Apr 26	-
Workplace closing	Mar 18–	Mar 12–Jun 1	-
Cancellation of public events	-	Mar 24–Jun 1	Mar 12–
Restrictions on gatherings	Mar 13–	Mar 12–	Mar 12–
Restrictions on gatherings to up to 10 pers.	Mar 18–May 7	Mar 24–Apr 20	-
Close public transport	-	-	-
Stay at home requirement	-	-	-
Restrictions on internal movement	-	Mar 16–Apr 19	-
International travel bans (specific regions)	Mar 11–	Mar 15–	Mar 19–
International travel bans (general)	Mar 14–May 24	Mar 15–Jun 14	-

We report only nationwide restrictions; recommendations are not reported. The table considers only the time period until June 30, 2020. An open end means that the measure was in place at least until June 30, 2020. Source: Oxford COVID-19 Government Response Tracker (Hale et al. 2020)

**Fig. 1:**
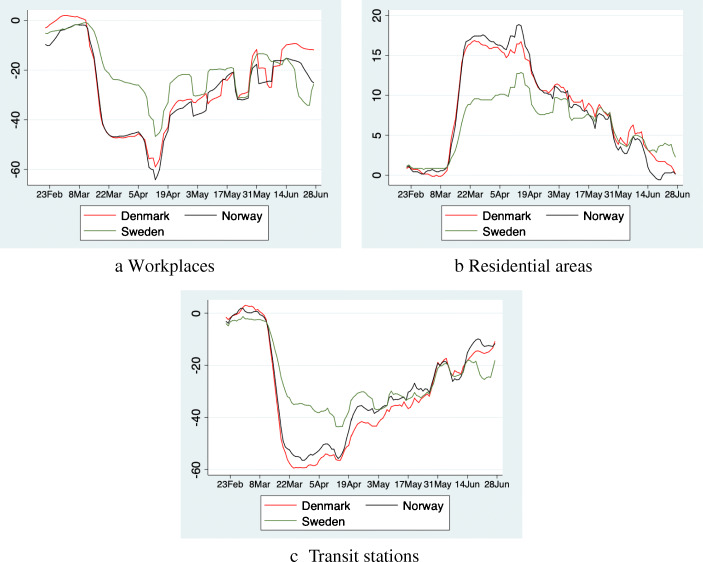
Activity in Denmark, Norway and Sweden compared to the baseline. The figure show how visits and length of stay at different places change compared to the median value, for the corresponding day of the week, during the 5-week period Jan 3–Feb 6, 2020. We present seven day moving averages to smooth weekend effects. Source: Google LLC “Google COVID-19 Community Mobility Reports”. https://www.google.com/COVID-19/mobility/, Accessed: July 1, 2020

The difference in restrictions is reflected in mobility data. Figure 1 shows that the initial reduction in mobility is roughly twice as strong in Denmark and Norway as in Sweden, whereas residential activity picked up strongly in Denmark and Norway relative to Sweden. This indicates that many workers and students worked or learned from home in Denmark and Norway. Note also, that mobility patterns in Norway and Denmark are virtually identical, owing to the fact that both countries passed very similar packages of NPIs.

Most of the restrictions in Denmark and Norway were phased out relatively rapidly after 4–5 weeks. This is also visible in the mobility data. It takes around 8 weeks for activity in Denmark and Norway to revert back to the Swedish level.

In our analysis, Sweden serves as a counterfactual to Denmark and Norway because it is the only country that has not initiated strict lockdown measures. It is therefore crucial to understand the similarities and differences between Sweden and the other two countries. Table 2 displays some key characteristics of Denmark, Norway and Sweden collected from the national statistics offices, Eurostat and Rhodes et al. (2012).

**Table 2:** Key country characteristics

	Denmark	Norway	Sweden
Population	5,827,463	5,367,580	10,327,589
Population density (per km^2^)	135.7	13.9	22.9
GDP per capita (in PPS)	126	150	121
Life expectancy at birth (years)	81.1	82.7	82.5
Healthcare spending (% of GDP)	10.2	10.5	11.0
Hospital beds (per 1mn inhabitants)	2,580	3,539	2,167
ICU beds (per 1mn inhabitants)	67	80	58
General medical practitioners (per 1mn inhabitants)	786	821	621
Nursing professionals (per 1mn inhabitants)	9,780	17,053	10,475
Self-reported unmet needs for medical examination (in %)	1.3	1.4	1.5
Share 67+ (in %)	17.6	15.4	17.9
Self-perceived health: Share of “bad”	5.8	6.6	4.3
Date first 100 cases	March 9	March 4	March 6
Cases until June 30 (per 1mn inhabitants)	2,191	1,656	6,755
Tests until June 30 (per 1mn inhabitants)	142,634	60,280	64,710

**Sources**: Population data 2019: National Statistics Offices. GDP 2018, Healthcare spending 2016, Life expectancy 2017, medical practitioners and nurses 2018, self-reported health statistics 2018, and hospital beds 2017: Eurostat. Intensive care beds 2011 includes intensive care and intermediate care beds: Rhodes et al. (2012). Cases and tests: Danish Health Authority (Sundhedsstyrelsen), Norwegian Institute of Public Health (Folkehelseinstitutt), Swedish Public Health Agency (Folkehälsomyndigheten) and C19.se

PPS: Eurostat’s Power Purchasing Standard in relation to the European Union average set to equal 100

Sweden has roughly twice the population of Denmark and Norway. In our analysis, to account for this difference, we consider only per capita measures of hospitalizations, ICU patients and deaths. In contrast, Denmark has a significantly higher population density. This is relevant, because in the absence of NPIs, epidemics are likely to spread faster in countries with higher population density. Therefore, in the case of Denmark, using Sweden as a counterfactual may underestimate the effectiveness of the NPIs. Norway has a lower population density than Sweden, and results may therefore be biased in the opposite direction.

All three countries have similar life expectancy and spend roughly the same percentage of their GDP on healthcare. However, Norway appears to have more medical capacity in terms of hospital beds, practitioners and nurses than the other two countries. Nevertheless, only between 1.3 and 1.5 percent of individuals in the three countries report that they have unmet needs in terms of medical examination. The similarities in the healthcare system have also been documented extensively in the medical literature (e.g., Kristiansen and Pedersen 2000; Lyttkens et al.^2016^).

Regarding, susceptibility to severe consequences of COVID-19, we find that Norway has the lowest share of the population over the age of 67 years, whereas Denmark and Sweden report about the same percentage. Conversely, Norway has the highest percentage of individuals who self-report being in poor health, while Sweden reports the lowest percentage.

Furthermore, we obtain data on the development of the ongoing COVID-19 pandemic from the national health authorities. The second panel of Table 2 shows the number of positively tested people, and the number of tests performed as of June 30, 2020.

Sweden has the highest number of confirmed cases per 1mn inhabitants at the end of our sample period. However, these numbers are difficult to compare because the countries implemented different testing policies. Denmark performed more than twice as many tests per capita than its peers. Even worse for a comparison is that the testing intensity also changed over time. For example, in the previous version of this paper, Norway performed the most tests per capita (Juranek and Zoutman 2020). This clearly shows that comparing countries on the basis of the number of reported cases is not meaningful in Scandinavia, which is why we do not include it among our outcome variables.

Finally, all three countries had winter school holidays roughly at the same time right before the first wave.^5^ These travellers are seen as an important channel for the initial infections (see, for instance, Brynildsrud and Eldholm (2020) for Norway). The timing of the winter holidays is roughly consistent between the three countries.

## Data

In our analysis we use three outcome variables. First, we use the daily number of patients with COVID-19 in ICUs (i.e., the number of patients with COVID-19 in ICU on a specific day in a specific country). Our second outcome variable is the daily number of patients in the hospital for COVID-19. Our final variable is the daily number of deaths related to COVID-19.^6^ Each outcome variable is normalized per 1 million inhabitants, and our sample ranges from March 5 to June 30, 2020. March 5 is our starting point, because it is the last day without COVID-19 hospitalizations and deaths in Scandinavia. Using these stock variables allows us to determine peak and overall pressure on the hospital sector.

Our analysis focuses on the narrow definition of Scandinavia, rather than including all Nordic countries. We chose to omit the island nations (Iceland, the Iceland Islands and the Faroe Islands), because we do not believe the spread of the virus in these islands would have been the same as the spread on the mainland in the absence of intervention. We also do not include Finland, because Finland does not provide data on hospitalizations and intensive-care admissions during the early weeks of the pandemic.

For Norway and Denmark, we use data from the national health authorities. Data is typically reported at a daily frequency. However, from June 6, 2020, onwards Norway no longer reports hospitalizations during the weekend. This does not affect our central estimates, unless the number of COVID-19 patients in the hospital is systematically different on weekends than on weekdays, which we consider unlikely. However, it does increase our standard errors for hospitalizations in Norway somewhat.^7^

For Sweden, we combine data from the national health authority with data from the Swedish aggregation website C19.se, which allows us to track the universe of COVID-19 mortality, and the number of hospitalizations and ICU patients from March 18th onwards.^8^ Prior to March 18, we have no information on the number of hospitalizations and ICU patients in Sweden, but the Swedish health authorities do report data on new patients admitted into ICU. From this data we gather that the first ICU admission in Sweden occurred on March 6. In our main analysis we assume that the number of patients in intensive care grows exponentially between March 6 and March 18. Since this assumption is crucial when we aim to verify common trends, we make two robustness tests in 7 in which we assume respectively i) linear growth rather than exponential growth, and ii) we use inflow data, which is available, and assume outflow from the ICU is a fixed percentage of the previous day’s number of patients in ICU. The details are presented in Appendix 7, but the main takeaway is that there is virtually no difference in the estimates.

For hospitalizations there is no way to interpolate Swedish data before March 18, and hence, our analysis for hospitalizations starts on March 18.

**Fig. 2:**
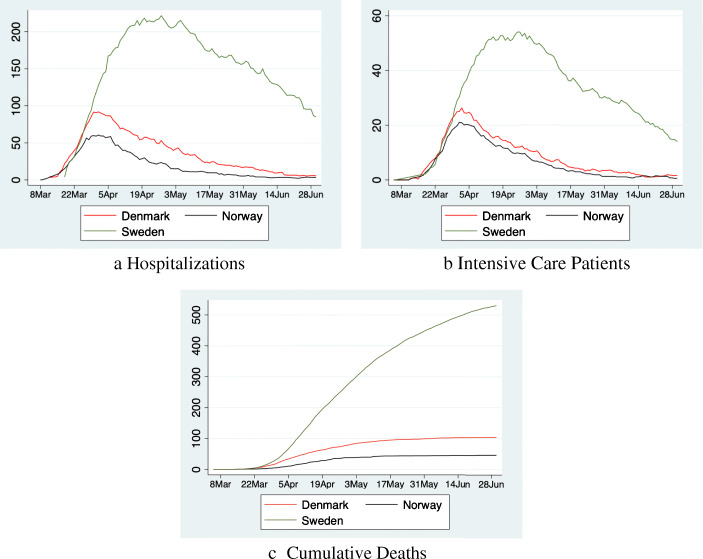
Number of patients hospitalized, in ICU and cumulative deaths per 1 mn inhabitants

Figure 2 shows our dependent variables. The number of hospitalizations and ICU patients initially follow a similar pattern in all three countries until somewhere between March 25 and March 30 (13–18 days into lockdown). After that Sweden continues reporting more hospitalizations and patients in ICUs, whereas the numbers in Denmark/Norway begin to plateau, and decrease which persists until the end of the sample period. In Sweden, numbers begin to decrease from May onwards, but remain at a much higher level. The number of COVID-19 deaths increases over time in all three countries, albeit at a much steeper rate in Sweden.

## Results

### Event-study

To quantify the effect of the lockdown we estimate an event-study model. We use daily data and estimate coefficients for each week (7 days) starting from March 5. For each week we calculate the DiD between Denmark/Norway and Sweden for our outcome variables using the week March 12–18 as our baseline period. The regression equation is given by:

1 $$ y_{ct\tau}= \beta_{c\tau} +\alpha_{c}+\gamma_{\tau}+\epsilon_{ct\tau},  $$

where *y*_*ctτ*_ denotes the outcome variable in country *c*, day *t* and week *τ*. *α*_*c*_ are country-fixed effects and *γ*_*τ*_ denote week-fixed effects. Our coefficient of interest is *β*_*cτ*_ which measures the DiD in the outcome variable between Denmark/Norway and Sweden and between week *τ* and the baseline period.

**Fig. 3:**
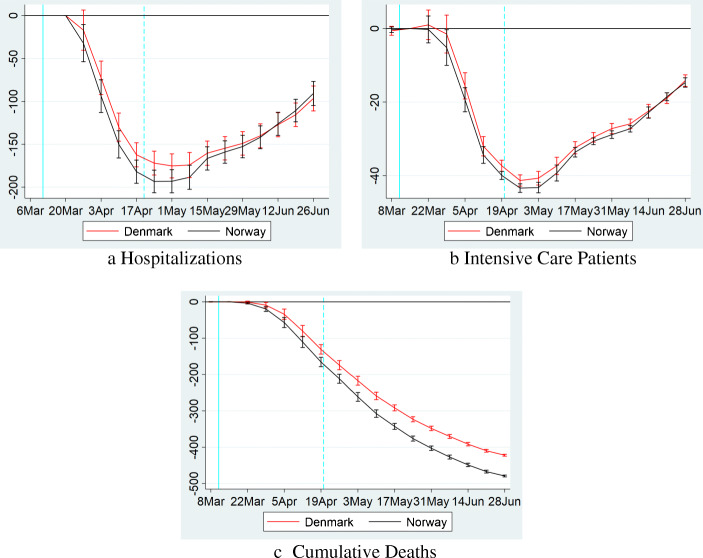
The effects of lockdown. The figure shows results from regression Eq. (1) for a the number of COVID-19 patients in hospitals, b the number of COVID-19 patients in ICUs, and **c** the cumulative number of deaths each per 1 million inhabitants. The coefficient for each week is shown on the fourth day of the period together with a 95-percent confidence interval, which we calculate using robust standard errors. The solid vertical line on March 11 represents the last day before Norway’s lockdown (Denmark’s lockdown occurred one day later). The dashed vertical line on April 19 represents the start of the phase-out in Norway (Denmark started phasing out on April 15). In panels **b** and **c**, the coefficient for the week of March 12–18 is normalized to 0. For panel **a**, the baseline period is March 18–24. The sample for panels **b** and **c** runs from March 5 to June 30. For panel **a**. the sample runs from March 18 to June 30

Results are presented in Fig. 3. The estimates in panels **b** and **c** show that in the initial weeks of the pandemic, patients in ICU, and mortality follow the same trend in all three countries.^9^ However, between 2 and 3 weeks into lockdown, patients in ICUs and mortalities start reducing in Denmark and Norway, relative to Sweden.

The timing of the effect is consistent with a causal effect from the lockdown. Specifically, the incubation period of COVID-19 from infection to symptoms is between 2 and 14 days with a median of 5 days (Lauer et al. 2020). Our outcome variables correspond to cases with severe symptoms which are likely to appear somewhat later than the initial symptoms. Hence, a causal effect of the lockdown is very unlikely to be measurable in the first week after lockdown which is consistent with our findings. The first measurable effect of the lockdown should instead occur 2–3 weeks after lockdown, and this effect should strengthen over time, since COVID-19 grows exponentially in the absence of lockdown. Both Denmark and Norway report negative coefficients two weeks into the lockdown (albeit initially not significantly so in Denmark), and the effect clearly grows with time.

Our results are also roughly consistent with Bonacini et al. (2020), who use machine-learning methods on Italian data to determine that NPIs start affecting COVID-19 cases between 17 and 19 days after they are introduced.

Another piece of evidence that we are really measuring a causal effect of the lockdown is how remarkably similar the pattern is in Norway and Denmark, consistent with the fact that both countries passed almost identical NPIs.

After the release of the lockdown, the DiD in ICU patients between Denmark/Norway and Sweden, begins to plateau, and subsequently reduce. It is important to note however, that this does not appear to be causally connected with the release of the lockdown. Instead, as can be seen in Fig. 2, the number of ICU patients in Denmark/Norway continues to decline (close to 0) even after restrictions are lifted, but the decline in Sweden is even larger. Deaths continue to decline in Denmark/Norway relative to Sweden, although at a less steep rate.

The phase-out of the NPIs likely did not result in an increase in COVID-19, because the release was rolled out slowly with emphasis on measures that prevent contagion.^10^

Hospitalizations follow a pattern similar to ICU patients. However, for hospitalizations we cannot analyze pre-trends before March 18.

Our main analysis excludes Finland. However, in Appendix 7 we replicate our event study for deaths using Finnish data. The main finding is that the DiD in deaths for Finland falls right in the middle between Denmark and Norway. This is consistent with the fact that Finland implemented roughly the same measures as Denmark and Norway (see Hale et al. 2020).

### Counterfactual analysis

To better understand the impact of the lockdown on hospital and ICU capacity, we use our model to make predictions on the peak number of COVID-19 patients in Danish and Norwegian hospitals in the counterfactual in which they would have followed Sweden’s more lenient social distancing approach. Our approach is as follows. First, we use the raw data to find the maximum number of patients in hospitals/ICUs in Denmark and Norway, and the date on which the peak occurs. Second, we find the counterfactual number of patients by predicting the number of patients in the absence of treatment, i.e., removing the treatment effect *β*_*cτ*_. Our counterfactual model is thus given by:

2 $$ y^{cf}_{ct\tau}= \hat{\alpha}_{c}+\hat{\gamma}_{\tau},  $$

where hats denote estimated values from regression Eq. (1).^11^

Results are reported in Table 3. Our model predicts that in the counterfactual without lockdown, Denmark (Norway) would have seen 103 (152) percent more patients in ICUs, and 144 (257) percent more hospitalizations at the peak. The peak would have occurred between 22 and 28 days later.

**Table 3:** Peak and cost analysis

		(1)	(2)	(3)
		Hospitalizations	ICUs	Cumulative deaths
	Peak date	Apr 1	Apr 2	NA
	Counterfactual peak date	Apr 23–29	Apr 23–29	NA
	Peak size	91.81	26.25	NA
	Counterfactual peak size	224.24	53.29	NA
	Relative difference	144.26%	102.96%	NA
Denmark	Patient-days/deaths	3,878.02	948.10	103.99
	Counterfactual patient-days/deaths	16,829.33	3,573.55	521.79
	Costs (in mn $)	9.5	23.2	872.6–3,490.6
	Counterfactual costs (in mn $)	41.3	87.4	4,378.6–17,514.4
	Relative difference	334.0%	277.0%	401.8%
	Peak date	Apr 1	Apr 1	NA
	Counterfactual peak date	Apr 23–29	Apr 23–29	NA
	Peak size	60.55	21.05	NA
	Counterfactual peak size	216.00	53.00	NA
	Relative difference	256.74%	151.74%	NA
Norway	Patient-days/deaths	2,070.02	739.63	46.76
	Counterfactual patient-days/deaths	15,963.84	3,539.41	521.45
	Costs (in mn $)	4.7	23.2	361.4–1,445.8
	Counterfactual costs (in mn $)	36.1	79.7	4,030.4–16,121.7
	Relative difference	671.19%	378.54%	1,015.11%

The table presents (i) results for the actual peak of patients in hospital/ICUs versus the model-predicted counterfactual peak in hospital/ICU patients, (ii) actual vs counterfactual number of patient-days in hospital/ICUs and deaths throughout the first wave, and (iii) calculations of the model-predicted benefits of the lockdown in terms of the number of patients/deaths, and in terms of cost. See the text for a description of the counterfactual model, and a justification for the cost parameter. Note that the peak date is provided in a 7-day window, since the predictions of the counterfactual model change every 7 days. The first panel shows results for Denmark, whereas the second panel shows results for Norway

We also calculate the overall effect of the lockdown on our outcome variables up to the end of the sample period. For patients in ICUs, and hospitalizations, we calculate ICU/hospital-patient days by summing the number of ICU/hospital-patients over the days in our sample period. Formally:

3 $$ Y_{c}=\sum\limits_{t=1}^{T} y_{ct\tau}. $$

To find counterfactual hospital/ICU-days we instead sum over the counterfactual outcome given by Eq. (2):

4 $$ Y_{c}^{cf}=\sum\limits_{t=1}^{T} y^{cf}_{ct\tau}. $$

For deaths, we simply consider the actual outcome, and the counterfactual outcome on June 30 at the end of the sample period.

Results are again reported in Table 3. We find that, without lockdown, Denmark (Norway) would have had 334 (671) percent more patient-hospital-days, 277 (379) percent more patient-ICU-days and 402 (1015) percent more deaths.

Finally, we calculate the overall benefit of a lockdown in terms of healthcare and mortality cost. We assume in our model that the cost per hospital/ICU-patient-day is fixed.^12^ We follow the WHO estimates for Denmark and Norway in 2005. We take the unweighed average and inflation correct them to 2019 USD. This results in a cost of 421 USD per patient per day.^13^

For the cost of a patient-day in an ICU, we follow Flaatten and Kvåle (2002) who estimate the average cost of critical care for ICU patients in a hospital in Norway. This paper provides one of the very few measures for the cost of ICU treatment in Scandinavia. They find, an ICU-patient costs 4,197 USD per day after inflation correction.

For the cost of mortality we consider a lower bound cost of 100,000 USD and an upper-bound of 400,000 USD per life-year.^14^ In accordance with Hall et al. (2020), we assume that on average a COVID-19 death results in a loss of 14.5 quality life years. We multiply the estimates with the population of Denmark/Norway in millions to get to aggregate results for both countries.

Overall, we find that Denmark (Norway) saved around 96 (94) million dollars in healthcare cost by locking down. The overall benefit of the lockdown amounts to 1.0–4.0 (0.9–3.5) percent of 2019 GDP for Denmark (Norway) depending on the cost per life-year. The large majority of costs is related to mortality.

## The long-term perspective

**Fig. 4:**
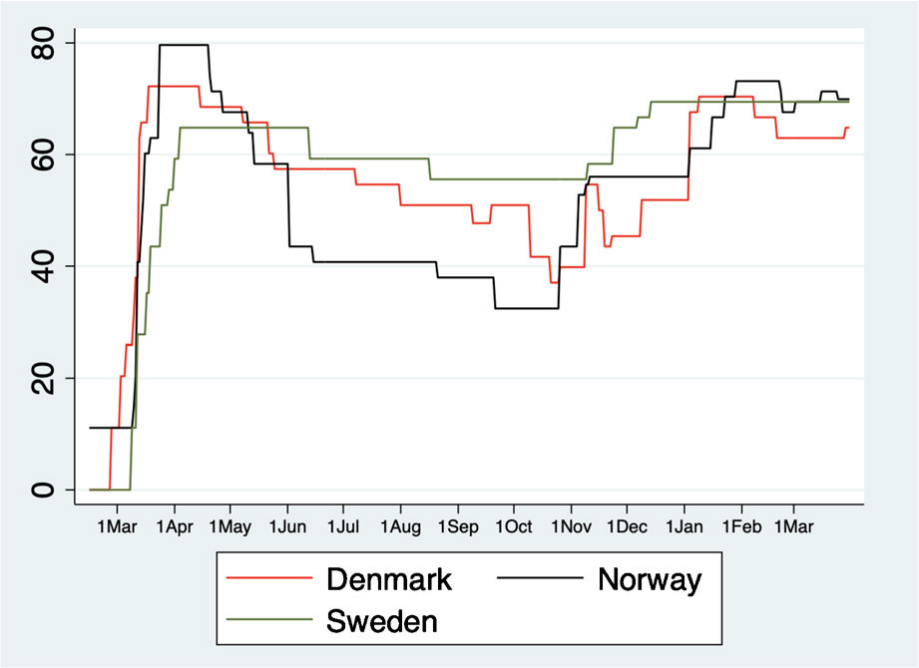
Stringency index over time. The figure shows the stringency index created by the Oxford COVID-19 Government Response Tracker (Hale et al. 2020) over time

In this section we consider what happened in Scandinavia after the first wave. Figure 4 shows the development of the stringency index created by the Oxford COVID-19 Government Response Tracker (Hale et al. 2020). The figure shows that NPIs in Sweden were less strict during the first wave. However, Norway and Denmark were able to ease the restrictions during the summer. In Norway, for example, international travel controls were loosened, workplaces were opened and even some public events took place. Daily life normalized to a wide extent. In Sweden, most of the restrictions remained in place.

During the second wave, all three countries tightened restrictions with fairly little policy variation between the Scandinavian countries. The level of restrictions is comparable to the initial lockdown in Denmark and Norway, and hence, much stricter than the NPIs Sweden introduced in the early phase of the pandemic.

It is, however, important to note that all countries learned from the first wave, and adjusted their strategies to their specific needs. For instance, the countries switched from a nationwide policy to a regional policy with the intention of containing outbreaks at the local level, before they spread nationally. At the same time the countries invested in a test-and-trace strategy, which is reflected in a strong increase in test capacity.

**Fig. 5:**
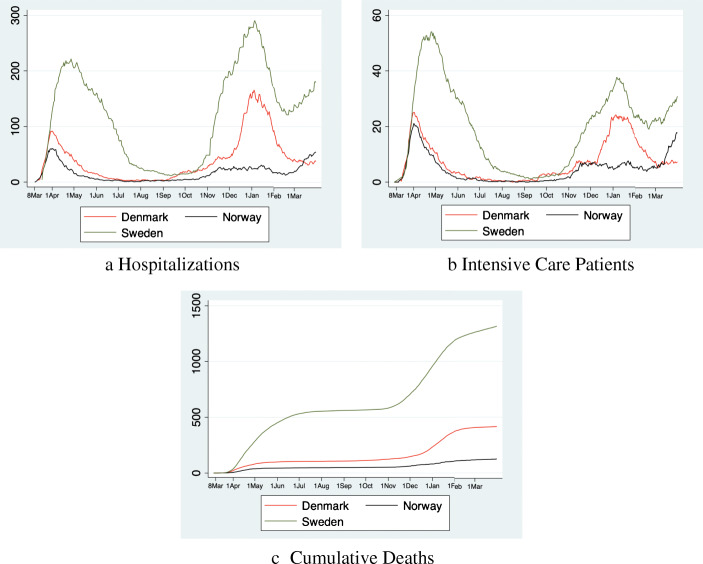
The long-term perspective. The figure shows the number of hospitalizations, intensive care patients and deaths per 1 million inhabitants until March 31, 2021

Turning to the developments in the healthcare system, we observe that in all countries the situation normalized during the summer (Fig. 5). However, the decline of the occupation of hospital and ICU beds took much longer in Sweden than in its two neighboring countries.

A second wave appeared in early fall, 2020, although Norway was mostly spared. Again, the situation was much worse in Sweden than in Denmark. After Christmas, all countries were able to decrease the pressure on the healthcare system and the second wave declined. However, in Sweden and Norway a third wave developed in early March 2021. The third wave remains absent in Denmark.

It is difficult to establish a causal connection between the measures and the spread of COVID-19 in the second and subsequent waves of the pandemic. Whereas we show that during the first wave, all three countries share a common trend prior to the initial lockdown in Denmark and Norway, such a common-trend assumption is not justifiable during the second, and subsequent waves. This probably also relates to the large decrease in international travel between the Scandinavian countries, which makes it far less likely that COVID-19 spreads quickly between the countries.

Nevertheless, looking at healthcare data it is clear that the Swedish strategy during the first wave did not result in long-term benefits. To the contrary, throughout 2020, and 2021 Sweden consistently has more COVID-19 hospitalizations, ICU admissions and deaths.

## Conclusion

Our study compares the effect on healthcare and mortality of the strict NPIs in Denmark and Norway with the more lenient approach of Sweden during the first wave of COVID-19. We show that the stricter measures decrease the stress on the healthcare system, and represent a significant benefit in terms of healthcare expenditure and mortality.

Overall, our results indicate that the lockdown in Denmark and Norway was successful, at least in the short and medium run. Relative to other countries, the strict lockdown was short, around five weeks. After release, there was no significant increase in hospitalizations and mortalities. On the other hand, data on mobility patterns in Fig. 1 shows that mobility in Denmark and Norway rapidly converges back to Sweden’s level after the lockdown, and takes about two months to fully reach it. This indicates that the Danish and Norwegian restrictions had a stronger effect than the Swedish recommendations.

The economic costs of the lockdown in Scandinavia in terms of consumer expenditure and employment have been well documented (Sheridan et al. 2020; Juranek et al. 2020) and remain relatively modest. For instance, Sheridan et al. (2020) find that consumer spending during the strict lockdown period dropped 4 percent more in Denmark than in Sweden. If we annualize this number, we end up with a $\frac {4 \%\times 5 weeks}{52 weeks}=0.38$ annualized percent drop in consumer spending, which is significantly smaller than our lowest estimate for the healthcare and mortality benefit.

The success of the lockdown is likely, in part, driven by the early date at which Denmark and Norway intervened (see, e.g., Amuedo-Dorantes et al. (2021), for the importance of the timing of NPIs). At the time of the lockdown, Denmark and Norway had less than 30 COVID-19 hospitalizations. It is well documented in the epidemiological literature that early interventions typically lead to better outcomes in epidemics (e.g., Hatchett et al. 2007).

Our analysis to some extent contrasts with the findings in the US that suggest that the fear of COVID-19, rather than restrictions is primarily responsible for the decrease in mobility (e.g., Goolsbee and Syverson 2021). Specifically, the Google Mobility data in Fig. 1 shows that during the lockdown, mobility in Denmark and Norway decreased substantially more than in Sweden despite the three countries having similar levels of COVID-19. This additional decrease in mobility is likely a prime contributor to the success of the lockdown.

There are several reasons why NPIs in Denmark and Norway might have been more effective at reducing mobility than those introduced in the US. First, it is well known that trust in the government and institutions is exceptionally high in Scandinavia (see, e.g., Rothstein and Stolle 2003), and Bargain and Aminjonov (2020) show that political trust leads to more compliance with NPIs. Second, the US may have suffered from conflicting messages across different political leanings whereas COVID-19 policy in Scandinavia was less politicized (see, e.g., Grossman et al. 2020). As a result it is likely that Scandinavians adhere to NPIs to a much greater extend than US residents.

A number of caveats are in order. First, our analysis only considers direct healthcare expenditure in hospitals. Therefore, we ignore primary care expenditure, cost related to absence from work, and the utility loss that results from suffering COVID-19. Second, most of the benefits of lockdown are the result of a reduction in mortality. Valuing the cost of mortality is controversial, and in part, a moral question. Here, we follow the consensus in the macro-economic literature, but policy makers may assign a different value to life, and hence come to a different policy conclusion.

With our data we can only compare the package of NPIs in Denmark and Norway to the package of NPIs in Sweden. Disentangling the effect of each NPI individually is not possible, since Denmark and Norway introduced all NPIs at roughly the same time. Identifying the effect of each NPI individually, requires pooling data from more countries, and using more sophisticated statistical modeling to distinguish between the causal effect of the NPI on the spread of COVID-19, and other omitted variables. We leave this as a venue for future research.

## Figures and Tables

**Fig. 6:**
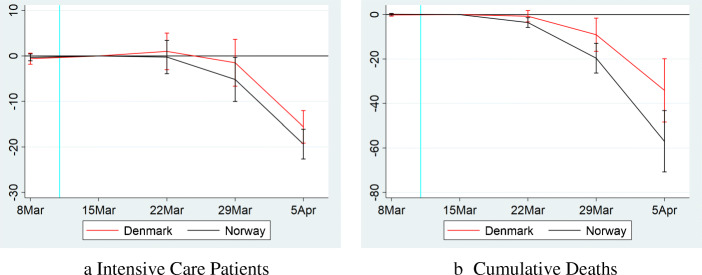
The effects of lockdown in the early weeks. This figure provides a zoomed in view of panel b and c of Fig. 3, focusing on the period up to April 5, 2020

**Fig. 7:**
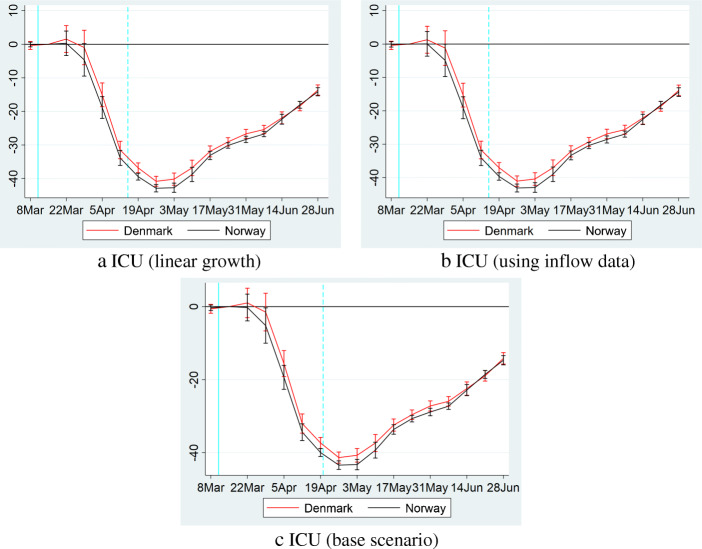
Common trends in ICU patients under different interpolations strategies for the early Swedish data. Panel a is similar to panel b of Fig. 3 but uses linear interpolation to determine the number of patients in Swedish ICUs between March 7 and 17 (Eq. (A2.1)). Panel b uses inflow data to interpolate Swedish ICU patients between March 7 and 17 (Eq. (A2.2)). Panel **c** is identical to panel b of Fig. 3, added for comparison

**Fig. 8:**
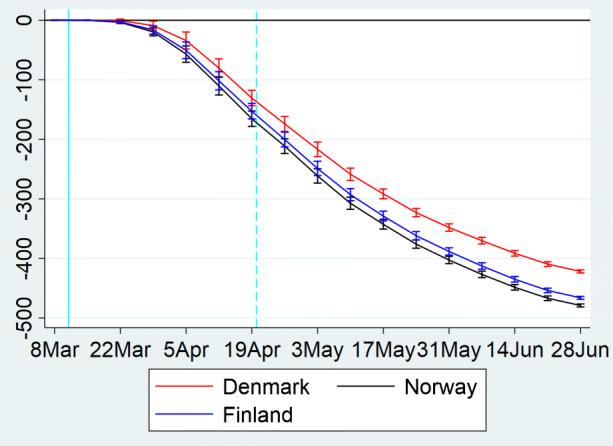
Deaths per 1 million inhabitants including data from Finland. The figure shows results from regression Eq. (1) for the number of cumulative deaths per 1 million inhabitants. The coefficient for each week is shown on the fourth day of the period together with a 95-percent confidence interval, which we calculate using robust standard errors. The solid vertical line on March 11 represents the last day before Norway’s lockdown (Denmark’s lockdown occurred 1 day later). The dashed vertical line on April 19 represents the start of the phase-out in Norway (Denmark started phasing out on April 15). The coefficient for the week of March 12–18 is normalized to 0. The sample runs from March 5 to June 30. Relative to panel c of Fig. 3, the difference is the inclusion of Finland

**Fig. 9:**
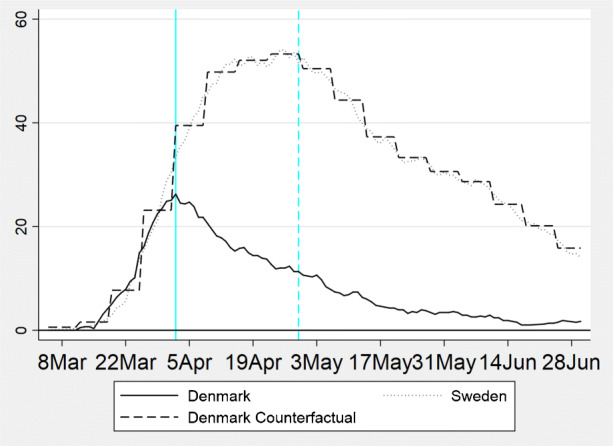
Graphical derivation of the peak results. The figure graphically depicts the derivation of our peak results in Table 3. The actual number of patients in ICUs in Denmark peaked on April 5th with 26 patients per 1 million inhabitants. The counterfactual numbers for Denmark follow from Eq. (2). They have a staircase pattern because in our event-study we estimate a coefficient for every week. This implies that the counterfactual changes on a weekly basis. The counterfactual peak occurs in the end of April. At that time, the model predicts there would have been 53 patients in ICUs per 1 million inhabitants, or around twice as many as in the actual peak. Note that the counterfactual of Denmark closely resembles Swedish data. The reason is that in the base-period Denmark and Sweden were similar in terms of both the trend, and the level of patients in ICUs

## Appendix 2. Alternative assumptions for interpolating the early ICU data in Sweden

As discussed in Section 2 of our paper, we do not have data on the number of patients in ICUs in Sweden between March 7 and March 17. In the paper we interpolate the missing data by assuming that the number of patients in ICUs between these two dates grows at an exponential rate such that:

$$ ICU_{Sweden,t}=\alpha ICU_{Sweden,t-1}. $$

Here we choose the multiplicative factor *α* to ensure the model-predicted number of patients on March 6th and March 18th coincides with the data (this implies *α* ≈ 1.26).

In this Appendix we explore two alternative assumptions to interpolate the data between March 7 and March 17. The first alternative assumption we make is that growth is linear. That is:

A2.1 $$ ICU_{Sweden,t}= ICU_{Sweden,t-1}+C,  $$

where *C* is chosen to ensure the model-predicted number of patients on March 6th and March 18th coincides with the data (this implies *C* ≈ 1.8).

Second, we use our data on ICU-patient inflow, which is available from March 5th onwards to predict the number of ICU patients between March 7 and March 17 as follows. By definition, we have that the number of ICU patients follows the process:

$$ ICU_{Sweden,t}=ICU_{Sweden,t-1}+Inflow_{Sweden,t}-Outflow_{Sweden,t}. $$

Therefore, for the period March 18–June 30 outflow can be found by inverting this expression:

$$ Outflow_{Sweden,t}=Inflow_{Sweden,t}-{\Delta} ICU_{Sweden,t}. $$

Using this relationship, we find the average percentage of the number of patients that flows out each day. We denote this average percentage by *γ*, and it equals around 6 percent in our sample for Sweden. We then predict the number of patients between March 7 and March 17 using:

A2.2 $$ ICU_{Sweden,t}=(1-\gamma)ICU_{Sweden,t-1}+Outflow_{Sweden,t}.  $$

Results are presented in Fig. 7. As can be seen, relative to panel b of Fig. 3 in the main text, there is only a marginal change in the coefficients.

## Footnotes

1 Note that we do not include Finland in our analysis. Finland does not provide hospitalization and intensive care data during the early weeks of the pandemic which is essential to our analysis. However, in Appendix 7 we include a robustness check in which we include Finnish data on deaths.

2 For hospitalizations our data starts on March 18th, and hence, we cannot establish pre-trends before that date.

3 There are three other papers that use Sweden’s exceptional approach to NPIs as a source of variation. Born et al. (2021) and Cho (2020) use the synthetic control method to compare Sweden to other European countries. A relative disadvantage of their approach is that they match on the number of cases, which is in part driven by time- and country-level variation in the testing regime, both of which are substantial. The analysis in Conyon et al. (2020) amounts to a replication of part of the analysis in earlier versions of this paper (Juranek and Zoutman 2020).

4 An important NPI not mentioned in the Table is testing and contact tracing. For instance, Salathé et al. (2020) describe the importance of contact tracing in the South Korean response to COVID-19. However, during the critical initial phase of the first wave testing criteria were very strict, and thus unlikely to have contributed much to containing the spread.

5 More specifically, Denmark had two weeks of winter holiday either in week 7–8 or 8–9 depending on the region. Norway had one week of winter holiday in either week 8 or week 9. Most of Sweden had one week of winter holiday in week 7–9. The only exception is Northern Sweden which had holiday in week 10 or 11 (but very few cases originate in the sparsely populated North of Sweden).

6 We observe the country-wide COVID-19 mortality. This implies we cannot distinguish between COVID-19 patients who died inside a hospital, and COVID-19 patients who died at home.

7 It should also be noted that by June 6th the number of COVID-19 hospitalizations in Norway is very low (12 times smaller than at the peak).

8 The national health authorities report the number of hospitalizations, and ICU patients from March 30th onwards. In the period from March 18 to 30 this data is only available on C19.se, which collects data from the Swedish Public Health Agency, the intensive care register, regional authorities, and the Swedish national television broadcaster (SVT). C19.se only reports data that has been confirmed by at least two sources. We confirm that after March 30, the data on c19.se coincides with the data published by the Swedish Public Health Agency.

9 Figure 6 in Appendix 7 provides a zoomed in view of the early week which allows for closer inspection of the common-trend assumption.

10 See for instance https://politi.dk/en/coronavirus-in-denmark/controlled-reopening-of-denmarkfor a full description of Denmark’s plan of releasing the NPIs

11 Appendix 7 provides a graphical explanation for how we arrive at our peak results for the example of the number of patients in ICUs in Denmark.

12 In reality there are intake costs associated with taking in new patients. As a result, the first day in the hospital is likely more costly than subsequent days (see, e.g., Dasta et al. 2005). Unfortunately, we cannot take this into account in our analysis, because we only have data on the number of patients for each day, and not on inflow and outflow.

13 Source: https://www.who.int/choice/country/US/cost/en/www.who.int/choice/country/US/cost/en/

14 These numbers are roughly based on Kniesner and Viscusi (2019) who consider the value of a statistical life-year used by the US government. They report a minimum vale of 116,000 USD in 1998 and a maximum value of 369,000 USD in 2016.
